# Oaks drought-induced responses under root types: gene and microRNA cooperation

**DOI:** 10.1186/s12870-025-07402-z

**Published:** 2025-10-01

**Authors:** Paulina Kościelniak-Wawro, Paulina Glazińska, Jacek Kęsy, Ewelina A.Klupczyńska, Joanna Mucha, Marcin Zadworny

**Affiliations:** 1 Institute of Human Biology and Evolution, Faculty of Biology, Adam Mickiewicz University, Uniwersytetu Poznańskiego 6, Poznań, 61-614 Poland; 2 Department of Ecology, Institute of Dendrology, Polish Academy of Sciences, Parkowa 5, Kórnik, 62-035 Poland; 3 Department of Plant Physiology and Biotechnology, Faculty of Biological and Veterinary Sciences, Nicolaus Copernicus University, Lwowska 1, Toruń, 87-100 Poland; 4 Department of Developmental Biology, Institute of Dendrology, Polish Academy of Sciences, Parkowa 5, Kórnik, 62-035 Poland; 5 Faculty of Forestry and Wood Technology, Poznan University of Life Sciences, Wojska Polskiego 71C, Poznań, 60-625 Poland

**Keywords:** Degradome, Drought stress, Gene expression, MiRNA, RNA-Seq, Transcriptome

## Abstract

Drought is a major stressor limiting plant growth and survival, requiring adaptive strategies. This study reveals that oak seedlings deploy distinct molecular and hormonal responses to drought depending on root type and cultivation method (containerized vs acorn-sown seedlings). Taproots and lateral roots exhibited divergent responses: taproots prioritized metabolic adaptation and sustained growth, while lateral roots emphasized growth restriction and structural adjustment. Seedlings initially grown in containers displayed unique transcriptomic and miRNA profiles compared to acorn-sown controls, reflecting the legacy of early root system constraints. Key regulatory networks involving stress-responsive genes, miRNAs, and hormones (notably ABA, JA, SA, and reduced auxins and cytokinins) underpin these adaptations. Our findings underscore the critical role of diversity within root types and cultivation techniques in enhancing drought resilience in oaks, and reveal often-overlooked molecular mechanisms that are vital to effective reforestation strategies aimed at climate mitigation and ecosystem restoration.

**Supplementary Information:**

The online version contains supplementary material available at 10.1186/s12870-025-07402-z.

## Background

Drought is a pervasive environmental stressor that significantly impacts the growth, development, and yield of agricultural and forestry crops [1, 2]. Rising global temperatures, driven by increased atmospheric CO₂ concentrations, coupled with reduced precipitation and more frequent extreme heat events, are expected to profoundly affect tree physiology and survival [3, 4]. However, the mechanisms underlying the differential drought tolerance among species remain poorly understood [5]. Some species exhibit superior drought avoidance, which may be attributed to their enhanced capacity for water acquisition, a trait more closely linked to root architecture and taproot development than to physiological adaptations conferring drought resistance [6]. From the perspective of functional plant traits taproots presence play a critical role in enhanced water access under drought [7–10].

At the root system growth scale, taproot (TR) and lateral roots (LR) organogenesis are governed by a combination of external and internal factors [11, 12]. Internal factors, including hormones, genes, and microRNAs, interact within complex regulatory networks to determine root size and function. The elongation of taproots is regulated by specific gene groups [13–15], which control lateral root establishment and the overall architecture of the root system [16]. Genetic factors governing root development begin influencing growth as soon as roots emerge from seeds and establish in the soil, shaping both individual root growth and overall root system architecture [13, 16]. These factors are also involved in maintaining undifferentiated cells in the root apical meristem (RAM), establishing root meristem identity, and ensuring root functionality [17]. Cell divisions in the RAM facilitate root elongation and deeper growth, increasing the root surface area available for water uptake. Conversely, under water-limited conditions, the excision of the RAM in primary roots promotes the formation of a highly branched, shallow root system [18–20]. This highlights the critical role of the RAM in defining root architecture [21, 22] and modulating water acquisition from deeper soil layers [23, 24]. These changes are coordinated by the expression of specific genes can trigger adaptive processes that enhance drought tolerance [25], including genes root elongation signaling to enhance their water-absorbing capacity [26, 27]. In oaks, taproot elongation and further water acquisition from deeper soil layer is one of the adaptive response to drought [24]. However, no studies have yet investigated how different seedling cultivation techniques influence the expression of genetic factors affecting taproot growth under drought stress, as taproot meristem may be damaged during oaks cultivation in the containers. How taproot growth response to water shortage is coordinated by drought responsive genes remains unclear.

Drought-responsive genes include those encoding heat shock proteins, glutamine transferases, dehydrins, proteinase inhibitors, and cytochrome P450s, among others [28]. Gene expression can be modulated by microRNAs (miRNAs), which inhibit the translation or induce the cleavage of target mRNAs. The interaction between miRNAs and their target mRNAs as well as miRNA play a pivotal role in tissue-specific gene expression and developmental regulation [11]. This balance is crucial for root development, particularly under drought stress, where disruptions can lead to partial or complete inhibition of root growth, as these molecules activate metabolic pathways regulating responses to abscisic acid (ABA), a key hormone in drought stress responses, as well as auxin signaling, osmoprotection, and antioxidant defense mechanisms [29, 30]. The regulation of these pathways determines growth capacity and stomatal control under drought stress. Despite their scientific and economic importance, the mechanisms driving differences in drought adaptation, including the activation or inhibition of drought-responsive regulators such as hormones, structural proteins, and root growth controllers, remain poorly understood. The synergistic networks between genes and other regulatory elements influence key drought-adaptive traits, such as increased primary root growth and a more vertical orientation of lateral roots, enabling deeper soil penetration [31]. Understanding these regulatory networks can provide insights into optimizing management practices to enhance tree resilience to climate change.

The limited knowledge on how miRNAs signaling of taproot origin define drought responses, hinders the development of science-based management strategies to enhance oak stand resilience to changing environmental conditions [32]. Even less is known about how taproots, whose growth is inhibited in containers and then resumes after transplanting into the rhizotron, respond to drought stress [10, 33]. In the face of rapidly changing climates and the growing use of containerized seedlings in forest regeneration, it is critical to determine whether oaks response to water shortage under shallow root distribution resulted from altered root architecture during containerized seedlings production or deep taproot formation of naturally developing oaks- acorn-sown, triggers the miRNAs signaling and further physiological response. To address these questions, we conducted RNA-seq analysis to profile transcriptome and miRNA sequences, identifying key molecules involved in drought response of acorn-sown oaks and containerized oaks. Additionally, given the pivotal role of plant hormones in drought stress responses, we quantified their endogenous levels under drought conditions.

## Materials and methods

### Plant material and cultivation

In this study, *Quercus robur* L. seedlings were cultivated using a rhizotron system, with acorns sourced from the National State Forests' storage facility in Jarocin, located within the Jarocin Forest District in Poland. The experiments were conducted in a large, semi-closed foil greenhouse at the Institute of Dendrology, Polish Academy of Sciences, in Kórnik, Poland [14]. Mean mass of single acorn used for sowing or containerized seedlings production was 4 g on average. Part of the acorns were sown in the transparent rhizotron chambers (30 × 50 cm). The rhizotron design featured two plexiglass plates spaced 2–3 cm apart and secured with durable plastic tubing, providing ample space for root development. The rest of acorns were sown in containers for containerized seedling cultivation. The containerized seedlings at Jarocin were grown in 18-cm deep by 50-mm wide containers with a volume of 0.275 dm^3^. We used peat and perlite mixture in a 5:1 volumetric ratio as growth substrates, supplemented with dolomite for deacidification and a slow-release fertilizer (Osmocote 15–9-12–2 N-P-K-Mg, including trace nutrients) at a concentration of 2.5 kg/m^3^ in rhizotron and container cases. Both oaks seedling cultivation techniques were cultivated for one year. In early spring of the following year one-year-old containerized seedlings were transplanted to the rhizotrons, without cutting the roots at transplanting time (containerized seedlings hereafter). This step was taken to simulate natural growth conditions, as seedlings from container nurseries are typically transplanted into the field after one year. Regardless of seedlings variant used from this point onward, watering system was installed in each rhizotron to regulate water supply, with the water supply tube positioned at the top of the rhizotron. Drainage systems were incorporated at the base of each rhizotron to prevent waterlogging. The use of rhizotrons allowed for non-invasive, longitudinal monitoring of root growth in the same seedlings without disturbing the root system.

### Water-shortage treatments

After 8-week acclimatization period when they grow under optimal watering conditions (April–June), one-year-old containerized oak seedlings that were transplanted into rhizotrons as well as one-year-old rhizotron seedlings were subjected to water shortage. The acclimatization period approach minimized the potential impact of varying growth conditions on changes in the plant transcriptome and miRNA. The timing of the drought treatment was aligned with the typical peak summer drought period. The drought stress was applied for 4 weeks (June – July). Watering was controlled using an automated irrigation system equipped with drip hoses connected to the each rhizotron. Since this time water supply at the bottom of the rhizotron depth of one-quarter from the bottom to encourage taproot growth, which extends deeper into the soil under conditions of water deficit in the upper soil layers and ensuring that water could only be accessed from the deeper areas of the rhizotron. Soil moisture was reduced to 25% of the levels in fully watered control plants by limiting water supply. Soil moisture in the rhizotron was monitored every two days at a depth of 15 and 45 cm in each rhizotron using moisture sensors, which automatically transmitted data to a data logger (SM150 Kit, Delta-T Devices). Temperature was measured on the soil surface using a HOBO Pro v2 monitoring system. The average air temperature recorded was 23.44 °C on the surface. The average air temperature at a height of 2 m was 20.48 °C.

Additionally, chlorophyll fluorescence in the leaves was measured during the drought experiment prior to harvesting using a Pocket PEA fluorimeter (Hansatech Instruments, United Kingdom) for both fully watered seedlings and those subjected to 4 weeks of 75% water restriction. We used daily chlorophyll fluorescence measurements in 20 seedlings of each cultivation system (rhizotron or containerized) and watering conditions (control and drought) since beginning of the water shortage treatment. We applied this measures to determine the point of severe drought stress in photosynthetic apparatus. Statistical significance was determined using Student's t-test. Significance levels are indicated as follows: * p ≤ 0.05, ** *p* ≤ 0.01, *** p ≤ 0.001. The measured parameters Fo/Fm (ratio of minimal to maximal fluorescence), Fv/Fm (maximum quantum efficiency of PSII), and Fv/Fo (ratio of variable to minimal fluorescence) are direct indicators of PSII (Photosystem II) efficiency. A decrease in these values under drought stress reflects damage to PSII, photoinhibition, and impaired functionality, respectively. The Performance Index (PI) provides a comprehensive measure of overall photosynthetic performance, and its decline indicates a severe reduction in the plant's ability to convert light energy into chemical energy. The final chlorophyll fluorescence measurements were conducted on the same day as the root material was collected for transcriptomic and miRNA analyses. This synchronization ensured that the physiological state of the plants, as reflected by the fluorescence parameters, directly corresponded to the molecular changes captured in the transcriptome and miRNA profiles. By performing these measurements concurrently, we were able to establish a clear link between the photosynthetic efficiency of the plants and their molecular responses to drought stress. This approach provided a comprehensive understanding of the plant's responses mechanisms under stress conditions.

### RNA isolation, library preparation and NGS sequencing

Samples were collected from the meristematic zones of taproots and lateral roots of both rhizotron and containerized seedling grown under two conditions: full irrigation (100% watering) and severe drought (75% moisture reduction). Each biological replicate consisted of 10–15 taproot tips containing the meristematic zone or lateral root tips emerging from the taproot. Total RNA was isolated using Total RNA Purification Kits and further purified with RNA Clean-Up and Concentration Kits (Norgen Biotek, Canada). Following isolation, RNA quality and quantity were assessed using a Nanodrop spectrophotometer and agarose gel electrophoresis. RNA integrity was evaluated using a Bioanalyzer 2100 (Agilent, United States), and only samples with an RNA Integrity Number (RIN) > 8 were selected for mRNA and miRNA library construction and > 7 for degradome sequencing.

### Transcriptome sequencing (RNA-seq)

cDNA libraries were prepared using the TruSeq Stranded mRNA LT Sample Prep Kit and sequenced on a NovaSeq 6000 platform (Illumina, San Diego, CA, USA) in 150 bp paired-end (PE) mode. Sequencing included mRNA molecules, with a sequencing depth of > 20 million reads per sample. All experiments were performed in triplicate to ensure reproducibility. For all analyses conducted, oaks sown directly into the rhizotron served as the reference point (background) for the studied taproot and lateral roots response of the containerized oaks to drought. The data generated in this study have been deposited in the NCBI Gene Expression Omnibus (GEO) and are accessible through GEO Series accession number GSE295319.

### miRNone sequencing (miRNA-seq)

Small RNA libraries were prepared using the TruSeq Small RNA Library Prep Kit (Illumina, San Diego, CA, USA) and sequenced on a NovaSeq 6000 platform in 50 bp single-end (SE) mode. The protocol specifically enriched for small RNAs in the 18–30 nt size range, with library quality verified by Bioanalyzer analysis. Each sample achieved a sequencing depth of > 20 million raw reads across triplicate biological replicates to ensure robust miRNA detection. The sequencing data captured comprehensive miRNA profiles from both taproot and lateral root meristematic zones of containerized versus directly sown oak seedlings under control and drought conditions. In all comparative analyses, rhizotron-sown oaks served as the reference baseline. The complete miRNA dataset has been deposited in the NCBI Gene Expression Omnibus (GEO) under accession number GSE295320.

### Degradome sequencing (RNA-seq RACE)

For degradome library preparation, approximately 20 μg of total RNA was used as starting material, with ~ 150 ng of poly(A) + RNA subsequently isolated from pooled samples of TR/LR-CT and TR/LR-DS and annealed to biotinylated random primers for first-strand cDNA synthesis, followed by streptavidin–biotin immobilization and specific ligation of a 5' adapter to RNAs containing 5'-monophosphates to enrich for cleavage products, which after reverse transcription and PCR amplification were sequenced on an Illumina HiSeq 2500 platform using single-end 50 bp reads focusing on the first 36 nucleotides to identify 5' RNA ends, employing this modified 5'-RACE approach (PARE/Degradome-Seq) based on Illumina's sequencing-by-synthesis technology to detect miRNA cleavage sites through their characteristic near-perfect base pairing with target mRNAs. The complete degradome sequencing dataset generated in this study has been deposited in the NCBI Gene Expression Omnibus (GEO) database and is publicly available under accession number GSE295322.

### Bioinformatics and functional analysis

*Transcriptome*: Raw RNA-seq reads obtained from transcriptome sequencing were trimmed and filtered to remove adapters using Cutadapt (https://cutadapt.readthedocs.io/en/stable/) [34]. Filtering was performed with a minimum read length threshold of 20 nucleotides. The trimmed reads were then mapped to the *Quercus robur* PM1N reference genome (https://www.oakgenome.fr/index8568.html?page_id=587) using HISAT2 (https://daehwankimlab.github.io/hisat2/) [35]. The quantification of mapped read pairs for individual genes was performed using HTSeq (https://htseq.readthedocs.io/en/release_0.10.0/index.html). [36], with strand-specificity (stranded or reverse) taken into account. Gene annotations were assigned based on InterProScan annotations.

To compare gene expression profiles between samples and identify genes with statistically significant differences in expression, we analyzed differentially expressed genes (DEGs) using DESeq2 for transcriptomes. DESeq2 employs a negative binomial distribution model, utilizing gene-specific means and dispersion parameters. The results of fold-change expression were tested using the Wald test, which provided information on the statistical significance of gene expression changes. The obtained *p*-values were adjusted for multiple testing using the Benjamini–Hochberg method. A false discovery rate (FDR) of ≤ 0.05 and a log2 fold change (log2FC) of ≥ 1.5 were used as thresholds to ensure high-quality data for the statistical analysis of DEGs. Results are presented as log2FC, representing the binary logarithm of the fold change in transcript abundance between the test and control samples.

In this study, a comparative analysis of the sequenced transcriptome was performed between the meristematic zone of taproots under control conditions (TR-CT) and drought stress (TR-DS), as well as lateral roots under control conditions (LR-CT) and drought stress (LR-DS). All up- and down-regulated genes are listed in Supplementary Table 1. All the genes presented in the table show statistical significance at p ≤ 0.05. The table contains key parameters from the RNA-seq analysis, including the average normalized counts per gene across samples (BaseMean), the magnitude and log2 scale of differential gene expression (Estimated_FoldChange and log2_Estimated_FoldChange), the Wald test statistic evaluating fold change significance (Wald_stat), raw and FDR-adjusted *p*-values accounting for multiple testing (*p*_value_not_adjusted and FDR_adjusted_*p*_value), and the standard error of the log2 fold change estimate (Standard_error_estimate). Additionally, it provides genomic context for each gene with chromosome location, gene type, start and end positions, and strand information (Chromosome, Type, Sequence_start, Sequence_end, Strand).

Gene Ontology (GO) and KEGG identifiers overrepresented in the set of differentially expressed genes (DEGs) were identified for each comparison. Only genes with an adjusted *p*-value below the conventional significance threshold of α = 0.05 were considered significantly differentially expressed. To identify GO terms significantly enriched among DEGs, the topGO package in R was used. For the identification of statistically overrepresented KEGG pathways in the DEG sets, the ClusterProfiler package.

(https://bioconductor.org/packages/devel/bioc/vignettes/clusterProfiler/inst/doc/clusterProfiler.html) [37] was employed, using the *Quercus lobata* genome (GCF_001633185.2) available in the KEGG database and its corresponding NCBI Entrez identifiers.

*miRNAs*: Similar to the transcriptome sequencing analysis, raw miRNA reads were trimmed using Cutadapt https://cutadapt.readthedocs.io/en/stable/ [34]. Filtering was performed with a minimum read length threshold of 15 nucleotides for miRNA. Quality control of the reads was conducted using FastQC (https://www.bioinformatics.babraham.ac.uk/projects/fastqc/). Subsequently, ShortStack was employed, which utilizes Bowtie (https://bowtie-bio.sourceforge.net/index.shtml) [38] for read mapping, as well as for the identification and quantification of miRNA molecules. For this analysis, all known miRNA sequences from the miRBase database were used.

Differential expression analysis of miRNA was performed using the edgeR package (https://bioconductor.org/packages/release/bioc/html/edgeR.html) [39] for the analyzed comparisons. A reference genome containing coding sequences (CDS) was used for this purpose. Potential target sites for each newly identified miRNA and mature miRNA from miRBase were detected using miRanda (with settings: score ≥ 150, energy ≤ −20 to minimize false positives) [40]. These sequences were matched against the reference genome containing coding gene sequences. Sets of target genes for significantly differentially expressed miRNAs were created for each comparison.

Gene Ontology (GO) and KEGG identifiers overrepresented in the set of target genes for significantly differentially expressed miRNAs were identified. Using the results from the previously conducted RNA-seq analysis, GO identifiers for target genes and EntrezId identifiers required for KEGG analysis were utilized. Only miRNAs with a false discovery rate (FDR) below the conventional significance threshold of α = 0.05 were considered significantly differentially expressed. The topGO package in R was used to assign GO identifiers with increased frequency among target genes of significantly differentially expressed miRNAs (FDR ≤ 0.05) [41]. The selected genes were characterized by Gene Ontology (GO) terms and classified into plant-specific GO categories: biological process (BP), cellular component (CC), and molecular function (MF).

Additionally, the ClusterProfiler package was used to identify statistically overrepresented KEGG pathways for target genes of significantly differentially expressed miRNAs with EntrezId identifiers [37, 42]. KEGG analysis was performed only for statistically significant miRNAs (FDR ≤ 0.05) from miRBase that had EntrezId identifiers.

In this study, a comparative analysis of sequenced miRNAs was conducted, encompassing the meristematic zone of taproots under control conditions (TR-CT) and drought stress (TR-DS), as well as lateral roots under control conditions (LR-CT) and drought stress (LR-DS). All up- and down-regulated miRNAs are listed in Supplementary Table 2.

*Degradome*: For the analyzed experimental variants, a bioinformatic analysis of the degradome was performed using CleaveLand4 (https://github.com/MikeAxtell/CleaveLand4/) [35]. To identify miRNA targets, the reference transcriptome of *Quercus robur* PM1N, used as the reference genome in the transcriptome analysis, was employed, along with miRNA sequences detected in the small RNA analysis.

### Validation of gene expression by RT-qPCR

The expression of selected genes identified through NGS sequencing was validated using RT-qPCR. cDNA was synthesized from 1 μg of total RNA or using SuperScript III Reverse Transcriptase (Invitrogen, Carlsbad, CA, USA), following the manufacturer’s instructions. oligo(dT) primers were used for first-strand synthesis. RT-qPCR reactions were performed on a LightCycler 480 (Roche, Switzerland) using KAPA SYBR® FAST qPCR Master Mix (Roche, Switzerland) and PCR primers listed in Supplementary Table 3, following the manufacturer’s protocols. All experiments were performed in triplicate to ensure reproducibility. Eight genes were used for validation (*NCER1* – neutral ceramidase 1; *WRKY* – WRKY transcription factor; *GST* – glutathione S-transferase; *PIN2* – auxin efflux carrier component 2; *GERL1* – germin-like protein 1; *ACO5* – 1-aminocyclopropane-1-carboxylate oxidase 5; *AIR1* – lipid-binding protein AIR1; *HK1* – hexokinase-1-like). *Elongation factor* (*EF*) was used as a reference gene.

### Plant hormone analysis

In our study, we analyzed the levels of the following plant hormones in both taproots and lateral roots under control conditions and drought stress: IAA (indole-3-acetic acid), IBA (indole-3-butyric acid), IBA (indole-3-butyric acid), tZ (trans-zeatin), 2iP (isopentenyladenine), GA3 (gibberellin 3), ABA (abscisic acid), JA (jasmonic acid) and SA (salicylic acid). These hormones were selected due to their critical roles in regulating plant growth, development, and stress responses. By comparing their levels in taproots and lateral roots under both control and drought conditions, we aimed to elucidate how hormonal regulation contributes to the plant's response to water deficit. This analysis provided insights into the complex hormonal networks that coordinate root growth, stress signaling, and resource allocation under drought stress. The analysis of plant hormone levels was performed using the QuEChERS method (quick, easy, cheap, effective, rugged, and safe) with deuterated internal standards, as described by [13]. Briefly, this method involves phase separation using acetonitrile and phytohormone extraction on C18 SPE columns (BAKERBOND Octadecyl spe™, Avantor, USA) to selectively bind and elute target compounds. Plant material preparation followed the protocol by Pu et al., with modifications. Hormone concentrations were calculated per gram of fresh weight [43, 44]. This approach enables the simultaneous analysis of hormone concentrations and gene expression from a single sample. It also minimizes errors arising from drought-induced accumulation of osmoprotectants, such as proline, which can distort dry weight-based measurements. Quantification was carried out using UHPLC-MS/MS with a Shimadzu Nexera XR UHPLC system coupled to a triple quadrupole mass spectrometer detector. Chromatographic separation was achieved using an Ascentis Express C18 column (2.7 μm, 100 × 2.1 mm) maintained at 35 °C. The mobile phases consisted of 0.1% formic acid in water (A) and methanol with 0.1% formic acid (B), with a flow rate of 0.35 mL min⁻^1^. The gradient program started at 35% B, increased to 90% B over 4 min, and then to 100% B over 2 min. Phytohormones were identified using multiple reaction monitoring (MRM). Data analysis was performed using LabSolutions 5.8 software, with log10 transformation applied to ensure normal distribution. Outliers were replaced with the average of valid measurements. Student’s t test applied to assess mean differences between taproot and lateral roots under control and drought conditions (p ≤ 0.05). All statistical analyses were conducted using JMP Pro.

## Results

### Drought stress severely impairs photosystem II efficiency and photosynthetic performance

The following parameters were analyzed to assess the efficiency of Photosystem II (PSII) and overall photosynthetic performance: Fo/Fm, Fv/Fm, Fv/Fo, PI (Performance Index), TRo/RC (trapped energy flux per reaction center), ETo/RC (electron transport flux per reaction center), ABS/RC (absorbed energy flux per reaction center), and DIo/RC (dissipated energy flux per reaction center). These parameters can be categorized into three main groups based on their functional roles: (1) PSII efficiency, (2) energy trapping and electron transport, and (3) energy absorption and dissipation (Table 1). Student’s t test applied to assess mean differences between the measured parameters under control and drought conditions (p ≤ 0.05). We indicated a decrease in Fo/Fm, Fv/Fm, and Fv/Fo reflecting damage to PSII and photoinhibition rise. Observed decline in the Performance Index (PI) provides a indicates a severe reduction in the plant's ability to convert light energy into chemical energy. Impaired energy trapping and disrupted electron transport was confirmed by reduction of TRo/RC and ETo/RC under water shortage conditions. We also observed inefficient utilization of absorbed energy due enhanced ABS/RC as well as energy loss in increased DIo/RC during drought stress (Table 1). Collectively, these parameters provide a detailed picture of photosynthetic disruption during drought stress, highlighting damage to PSII, impaired electron transport, and increased energy dissipation.

**Table 1:** The effect of drought stress on Fo/Fm—minimal fluorescence/maximal fluorescence; Fv/Fm – maximum quantum yield of PSII; Fv/Fo – variable fluorescence/minimal fluorescence; PI – performance index; TRo/RC – trapped energy flux per reaction center; ETo/RC – electron transport flux per reaction center; ABS/RC – absorption flux per reaction center; DIo/RC – dissipated energy flux per reaction center of leaf of oak seedlings in comparison to control conditions. Mean values are presented and standard deviations are put into the brackets

Treatment	Fo/Fm	Fv/Fm	Fv/Fo	PI	TRo/RC	ETo/RC	ABS/RC	DIo/RC
Control	0.81 (0.02)	0.80 (0.02)	4.52 (0.5)	4.25 (0.5)	2.81 (0.2)	1.86 (0.1)	2.33 (0.2)	0.80 (0.1)
Drought	0.65 (0.04)*	0.68 (0.05)*	2.41 (0.5)***	0.83 (0.2)***	1.24 (0.3)***	0.87 (0.2)**	3.56 (0.4)**	3.26 (0.5)***

Means of drought and control treatment marked with asterisks differ statistically: * p ≤ 0.05, ** *p* ≤ 0.01, *** *p* ≤ 0.001 (t-Student test)

### Drought stress induces distinct and overlapping transcriptomic responses in taproots and lateral roots, highlighting metabolic and hormonal adaptations

**Fig. 1:**
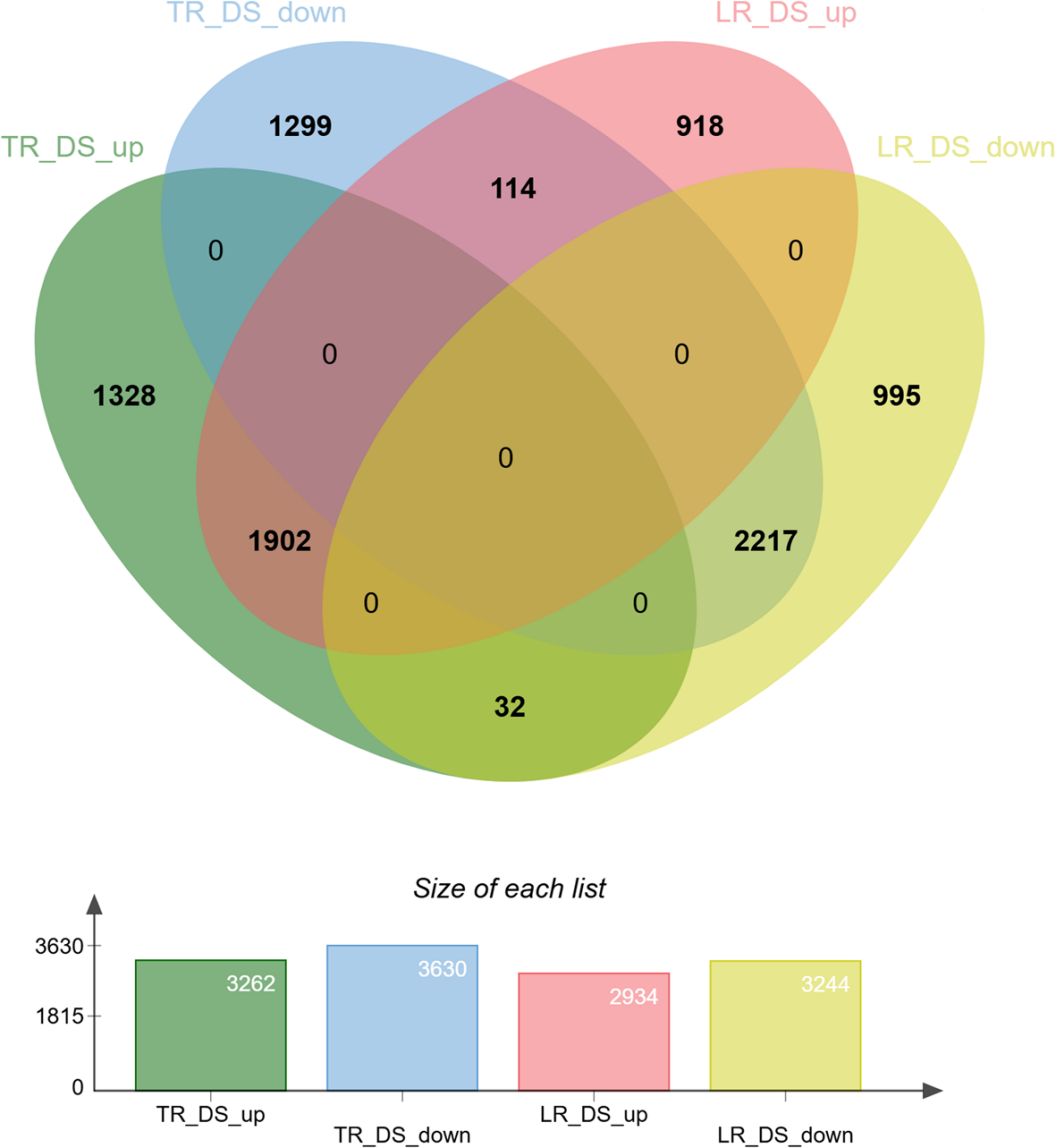
Venn diagram illustrating up- and down-regulated genes in taproots and lateral roots during drought stress compared to the control without drought stress. TR – taproot, LR – lateral roots, DS – drought stress, up – up-regulated genes, down – down-regulated genes

Differential expression genes (DEG) analysis revealed 3,262 up-regulated and 3,630 down-regulated genes in taproots subjected to drought stress (TR-DS) compared to the control. In lateral roots under drought stress (LR-DS), 2,934 genes were up-regulated and 3,244 were down-regulated relative to the control (Fig. 1). Among these, 1,902 genes showed increased expression, while 2,217 genes exhibited decreased expression in both TR-DS and LR-DS. Additionally, 114 genes were down-regulated in TR-DS but up-regulated in LR-DS under drought stress, whereas 32 genes were up-regulated in TR-DS and down-regulated in LR-DS.

Analysis of up-regulated genes in TR-DS and LR-DS highlighted the presence of numerous genes associated with drought stress response and plant hormone regulation. These included genes involved in cellular dehydration protection, hormonal regulation, metabolism and detoxification, signal transduction, carbohydrate and cell wall metabolism, transport, oxidative stress response, cell wall modification, pathogen response, carbohydrate metabolism, and transcriptional regulation.

**Fig. 2:**
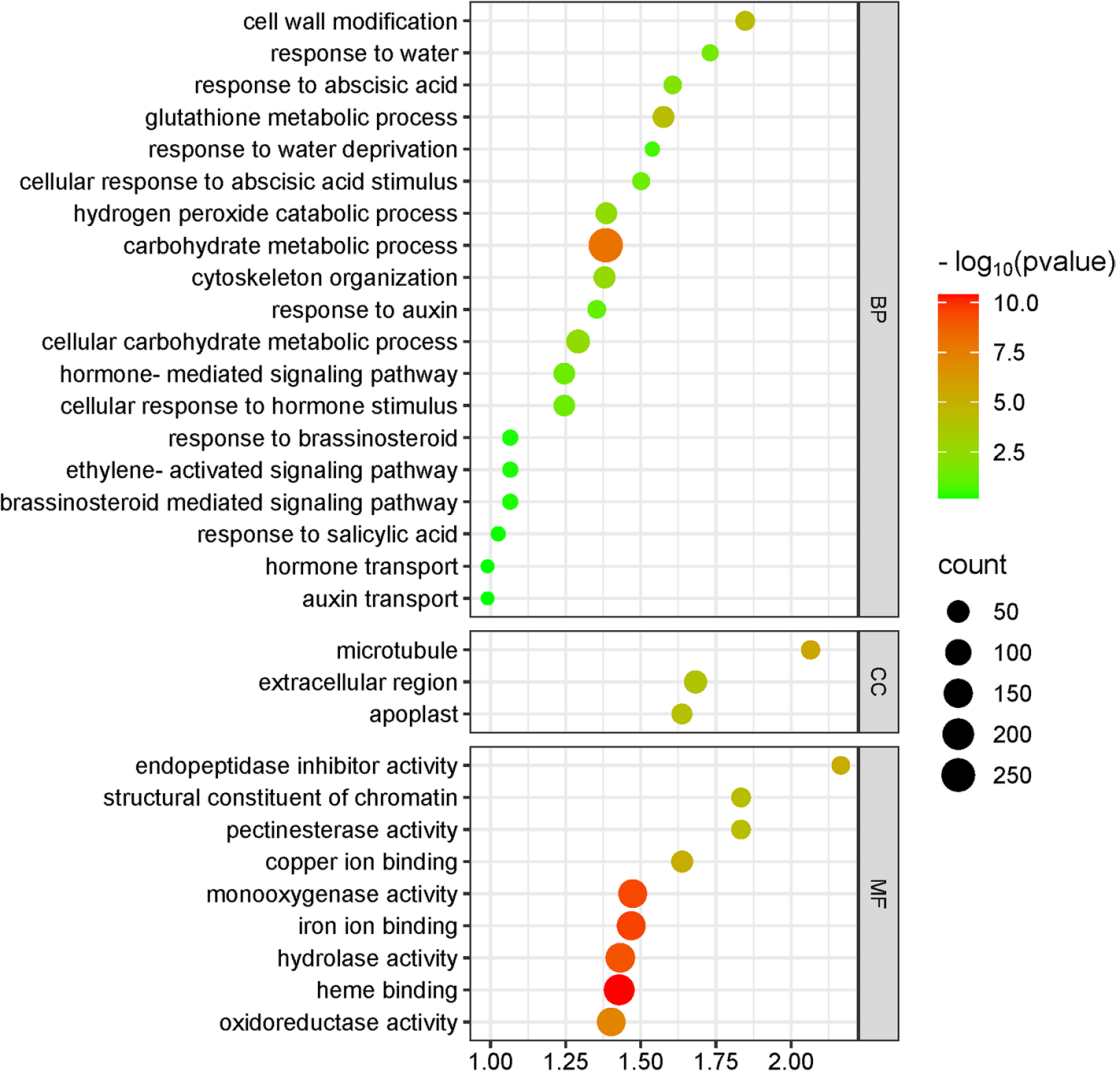
GO term enrichment analysis in taproot during drought stress. The figure illustrates KEGG metabolic pathways, with each circle symbolizing a specific pathway. The size of each circle reflects the number of genes enriched in that pathway. The significance of the enrichment of differentially expressed genes (DEGs) within a pathway is represented by -log10 (*p*-value). Pathways with low q-values are shown in green, while those with high q-values are depicted in red. Additionally, the size of the circle corresponds to the number of enriched genes in the pathway. BP – biological process; CC – cellular component; MF – molecular factor

GO analysis revealed that in TR-DS under drought stress in the biological process (BP) category the most highly expressed genes were associated with carbohydrate metabolic process, glutathione metabolic process, cell wall modification, and response to water deprivation. In the cellular component (CC) category, the most prominent terms were microtubule, extracellular region, and apoplast, while in the molecular function (MF) category, the top terms included heme binding, iron ion binding, monooxygenase activity, hydrolase activity, and oxidoreductase activity. Furthermore, many genes in the BP category were linked to responses to plant hormones, including abscisic acid, auxin, brassinosteroids, ethylene, and salicylic acid (Fig. 2).

**Fig. 3:**
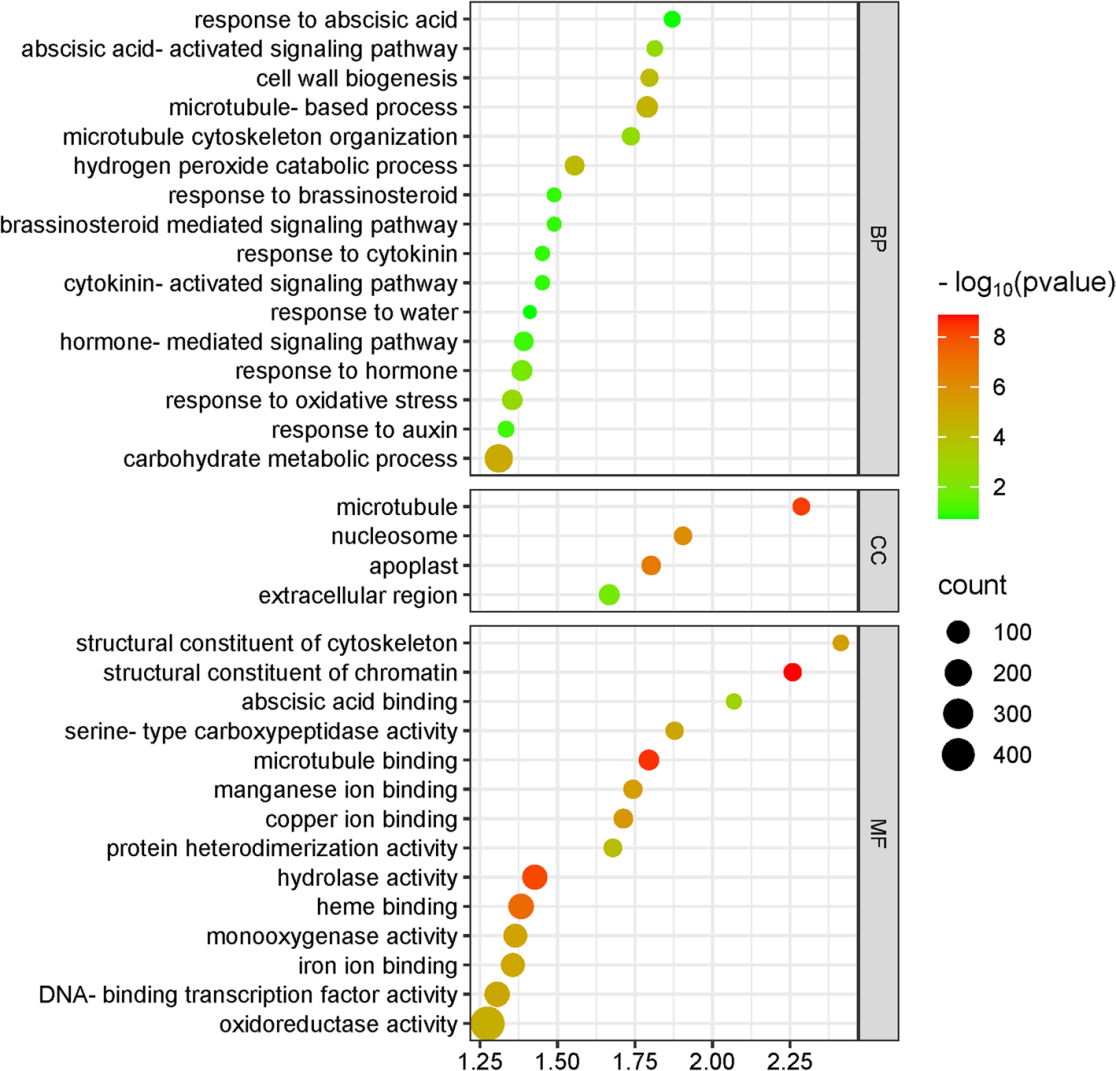
GO term enrichment analysis in lateral roots during drought stress. The figure illustrates GO metabolic pathways, with each circle representing a specific pathway. The size of each circle reflects the number of genes enriched in that pathway. The significance of the enrichment of differentially expressed genes (DEGs) within a pathway is represented by -log10 (*p*-value). Pathways with low q-values are shown in green, while those with high q-values are depicted in red. Additionally, the size of the circle corresponds to the number of enriched genes in the pathway. BP – biological process; CC – cellular component; MF – molecular factor

In LR-DS under water shortage, the BP category was dominated by genes involved in carbohydrate metabolic process, microtubule-based process, hydrogen peroxide catabolic process, and cell wall biogenesis. For the CC category, the most significant terms were microtubule, nucleosome, apoplast, and extracellular region, while the MF category included genes associated with structural constituent of chromatin, microtubule binding, hydrolase activity, heme binding, ion binding (copper, iron, manganese), structural constituent of cytoskeleton, monooxygenase activity, DNA-binding transcription factor activity, serine-type carboxypeptidase activity, oxidoreductase activity, protein heterodimerization activity, and abscisic acid binding. Similar to TR-DS, many genes in the BP category were related to responses to plant hormones, including cytokinins, auxin, brassinosteroids, and abscisic acid (Fig. 3).

**Fig. 4:**
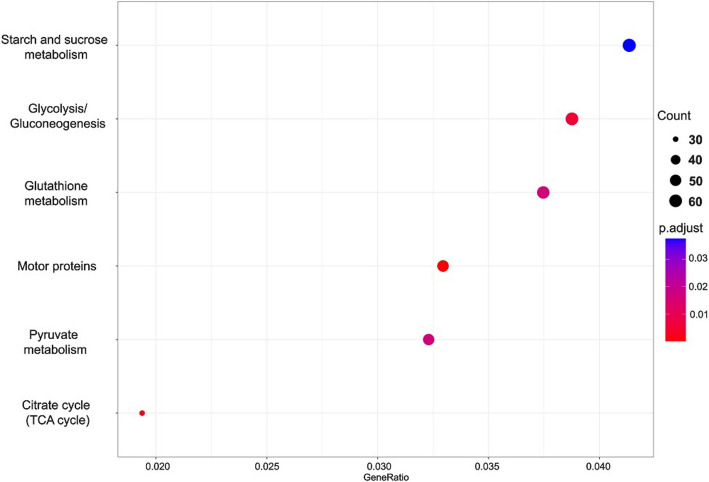
KEGG term enrichment analysis in taproot during drought stress. The results are displayed as p.adjust values, which represent statistical significance after applying a correction for multiple testing. A lower p.adjust value indicates greater statistical significance. Pathways with lower p.adjust values are highlighted in blue, while those with higher significance are shown in red

**Fig. 5:**
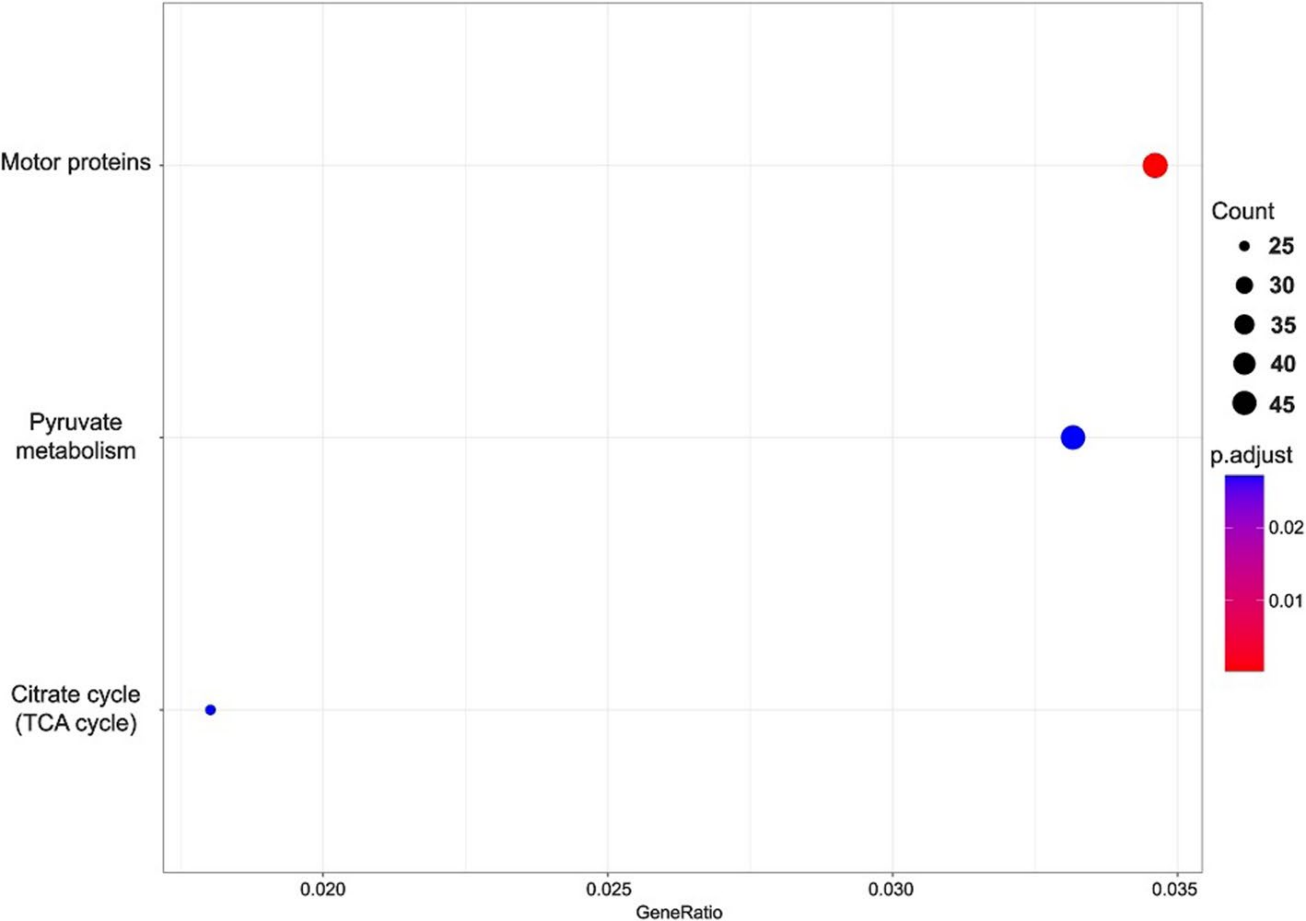
KEGG term enrichment analysis in lateral roots during drought stress. The results are displayed as p.adjust values, which represent statistical significance after applying a correction for multiple testing. A lower p.adjust value indicates greater statistical significance. Pathways with lower p.adjust values are highlighted in blue, while those with higher significance are shown in red

KEGG pathway analysis revealed significant enrichment of pathways related to starch and sucrose metabolism, glycolysis/gluconeogenesis, glutathione metabolism, motor proteins, pyruvate metabolism, and the citrate cycle (TCA cycle) in taproots subjected to drought stress compared to taproots grown under fully watered conditions (Fig. 4). In lateral roots under drought stress, significant changes were observed in pathways associated with motor proteins, pyruvate metabolism, and the citrate cycle (TCA cycle) (Fig. 5).

### Distinct miRNA expression patterns in taproots and lateral roots indicate tissue-specific regulatory strategies for drought adaptation

Sequencing of miRNA molecules in oak roots revealed their the lower abundance in taproots (58 mature miRNAs) in comparison to 61 mature miRNAs observed in lateral roots under drought stress conditions. Among the former, 35 known miRNAs and 23 novel miRNAs were identified in taproots subjected to drought stress (TR-DS), while in the latter root types 37 known miRNAs and 24 novel miRNAs were identified under drought stress (LR-DS). In TR-DS, 29 miRNAs were up-regulated and 29 were down-regulated, whereas in LR-DS, 24 miRNAs were up-regulated and 37 were down-regulated.

A total of 19 miRNAs were up-regulated in both TR-DS and LR-DS, and these are associated with root development and architecture (e.g., miR166a, miR166c, miR166d, miR171b, miR171d, miR160b, miR167b, and miR396a), stress responses (e.g., miR393a, miR398a, miR162a, miR403a), hormonal regulation (e.g., miR160b, miR167b, and miR393a), gene regulation (e.g., targeting transcription factors or key regulatory genes to modulate gene expression networks in response to stress and developmental cues; qrb-miR166a, qrb-miR166c, qrb-miR166d, qrb-miR171b, qrb-miR171d, qrb-miR162a, qrb-miR403a, qrb-miR396c), and oxidative stress management (e.g., qrb-miR398a). In contrast, 25 miRNAs were down-regulated in both TR-DS and LR-DS, and these are involved in processes such as stress responses (e.g., miR166b, miR171c, miR171e, miR319a, miR164c, miR395e, miR168, miR535a), developmental regulation (e.g., miR166b, miR171c, miR171e, miR319a, miR164c, miR396b, and miR535a), hormonal regulation (e.g., miR390b and miR159a), and gene regulation (e.g., targeting transcription factors or key regulatory genes to modulate gene expression networks in response to stress and developmental cues; miR166b, miR171c, miR171e, miR319a, miR164c, miR396b, miR168a, and miR535a).

**Fig. 6:**
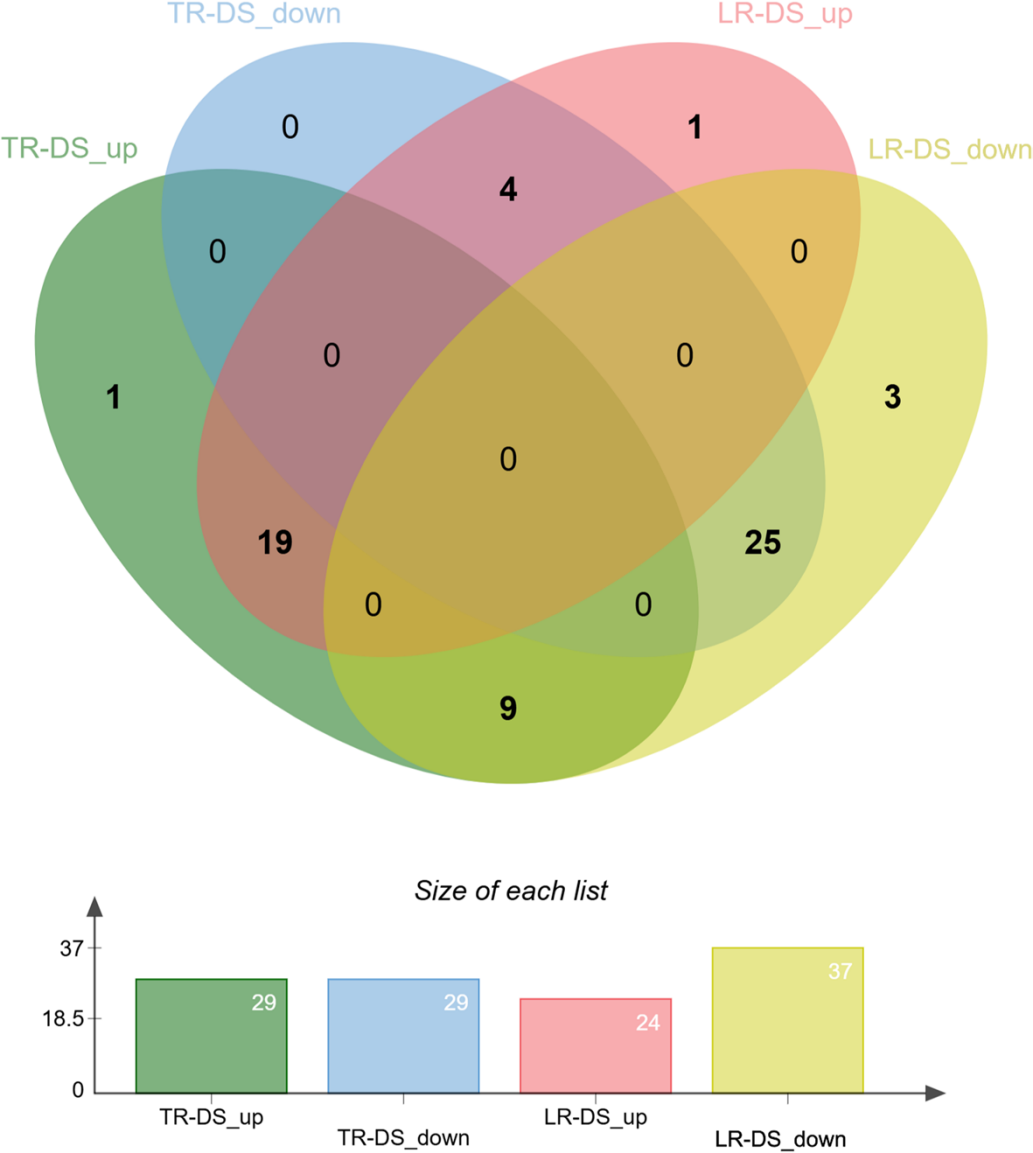
Venn diagram illustrating up- and down-regulated miRNA in taproots and lateral roots during drought stress compared to the control without drought stress. TR – taproot, LR – lateral roots, DS – drought stress, up – up-regulated genes, down – down-regulated miRNA

Novel miRNAs are likely involved in stress responses, gene regulation, and developmental processes, similar to known miRNAs, though their exact functions require further characterization. Noticeably, 9 miRNAs exhibited up-regulation in TR-DS including miRNAs that modulate root growth and stress adaptation by regulating transcription factors such as AP2-like transcription factors involved in developmental transitions and stress responses (miR172a), GRF (Growth-Regulating Factors) involved in cell proliferation and stress responses (miR396a), ARF (Auxin Response Factor) genes involved in auxin signaling and root development (qrb-miR167a), CSD (Copper/Zinc Superoxide Dismutase) genes involved in oxidative stress management (miR398b), NAC transcription factors involved in root development and stress responses (miR164d), genes involved in copper homeostasis and stress responses (miR408a), and TAS3-derived small RNAs, which target ARF genes involved in auxin signaling (miR390a). Interestingly, we observed simultaneously down-regulation of these miRNAs in lateral roots under drought stress (Fig. 6; Supplementary Table 4).

In LR-DS, we observed lower number of up-regulated miRNAs (4), including 2 novel miRNAs and 2 miRNAs of known function: miR171a, which regulates GRAS transcription factors involved in root development, stress responses, and hormonal signaling, and miR166e, which targets HD-ZIP III transcription factors crucial for root and shoot development and stress responses (Supplementary Table 4).

The miR166 family, in particular, exemplifies the complexity of miRNA-mediated regulation under drought. Our results showed divergent expression patterns among miR166 isoforms: while miR166a, miR166c, and miR166d were up-regulated in both TR-DS and LR-DS, miR166b was down-regulated in these treatments, and miR166e was up-regulated in LR-DS but down-regulated in TR-DS. This diversity can be differentially regulated and may target different subsets of HD-ZIP III transcription factors. In our study, miR166a and miR166d, which share identical mature sequences but are encoded by different loci, targeted the same HD-ZIP III genes (Revoluta and ATHB-15, confirmed by degradome analysis), suggesting coordinated regulation. In contrast, miR166b, with a distinct sequence, may be independently regulated and potentially targets different HD-ZIP III members or acts at the translational level, as it was not detected in our degradome data. The subtle expression changes of miR166e (logFC − 0.39 in TR-DS and 0.12 in LR-DS) indicate a marginal or context-specific role.

Overall, these findings highlight the functional diversification within miRNA families, such as miR166, enabling fine-tuned and tissue-specific regulation of root development and stress adaptation in oak under drought conditions. Further studies are needed to clarify the specific roles and mechanisms of individual miRNA family members in drought response.

### Distinct miRNA–target correlation patterns highlight complex post-transcriptional regulation in drought-stressed roots

**Fig. 7:**
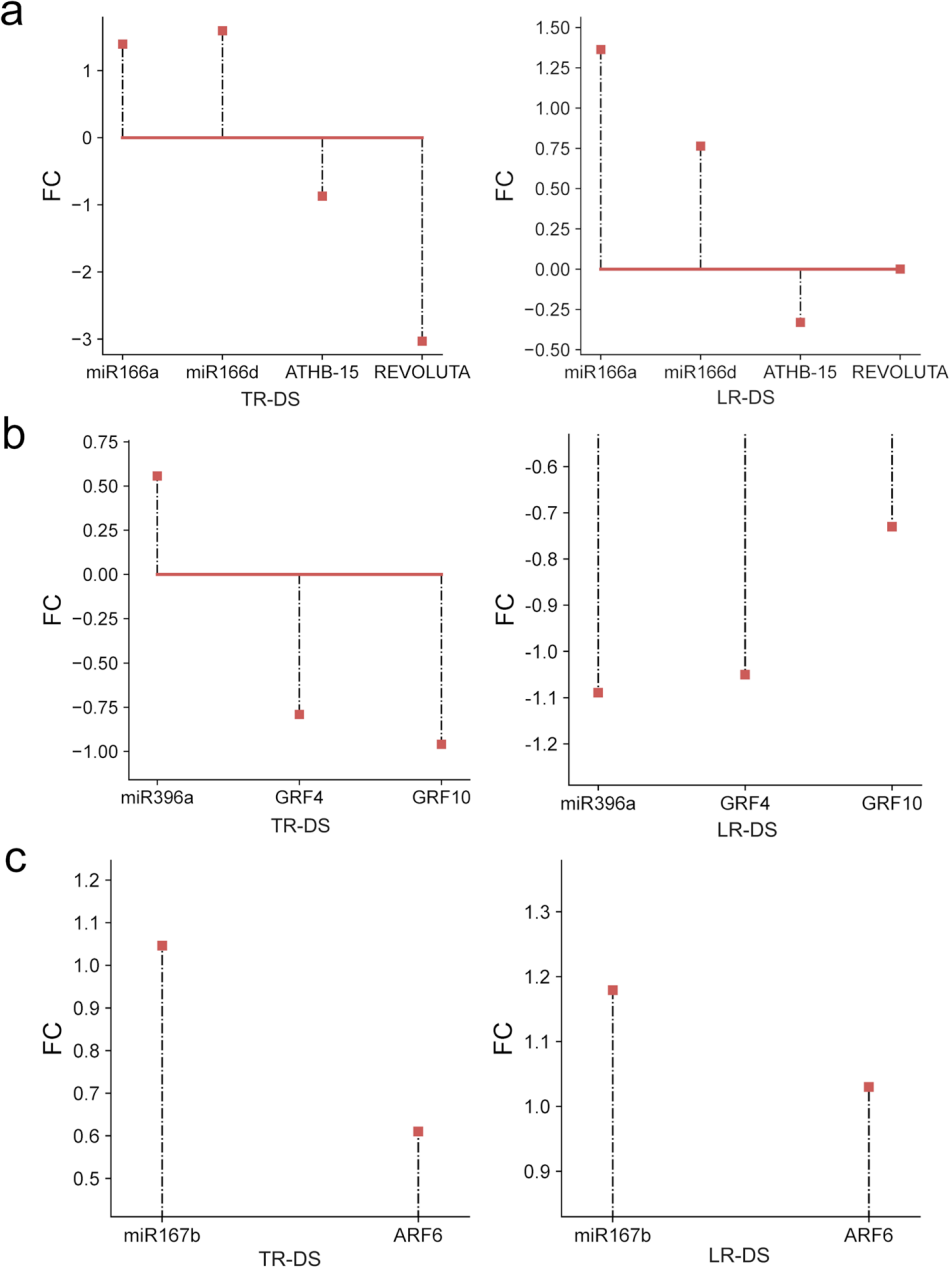
Comparison of the expression levels (FC) of miRNAs and their targets and in TR-DS and LR-DS as determined by deep sequencing (**a**) qrb-miR166a, qrb-miR166d and their targets gene Qrob_T0357230.2_ATHB-15 mRNA and Qrob_T0602040.2_ REVOLUTA mRNA, (**b**) qrb-miR396a and its target genes Qrob_T0026800.2_GRF4 mRNA and Qrob_T0769850.2_GRF10 mRNA, (**c**) qrb-miR167b and its target gene Qrob_T0190770.2_ARF6 mRNA

To define the biological function and impact of selected miRNAs, we identified their target genes. This method has revealed numerous known and novel miRNA targets in plants. In our study, degradome sequencing yielded at least 10,460,321 raw reads per library. After quality filtering, the reads were aligned to the reference transcriptome using CleaveLand 4 to identify sliced mRNAs. Following additional processing and filtering with a significance threshold of p < 0.05, between 33 and 45 targets per library were identified (Supplementary Table 5). A full list of degradome-identified targets is provided in Supplementary Table 6. Exemplary target plots (T-plots) showing miRNA–mRNA interactions are presented in Supplementary Fig. 1–5. The T-plots show the distribution of the degradome tags along the full length of the target gene sequence. The cleavage site of each transcript is indicated by a red dot. A comparison of the expression levels of five exemplary miRNAs and their corresponding target genes (Fig. 7) indicates that qrb-miR166a, qrb-miR166d, and their targets — Qrob_T0357230.2 (ATHB-15) and Qrob_T0602040.2 (REVOLUTA) — exhibit a classical reverse correlation between miRNA accumulation and mRNA abundance. In the case of qrb-miR396a and its targets — Qrob_T0026800.2 (GRF4) and Qrob_T0769850.2 (GRF10) — a reverse correlation was observed in the taproot (TR). In lateral roots (LR), both the miRNA and its target genes were down-regulated under drought stress (DS). For qrb-miR167b and Qrob_T0190770.2 (ARF6), both molecules were upregulated under DS.

### RT-qPCR validation confirms reliability of RNA-seq data in oak roots

**Fig. 8:**
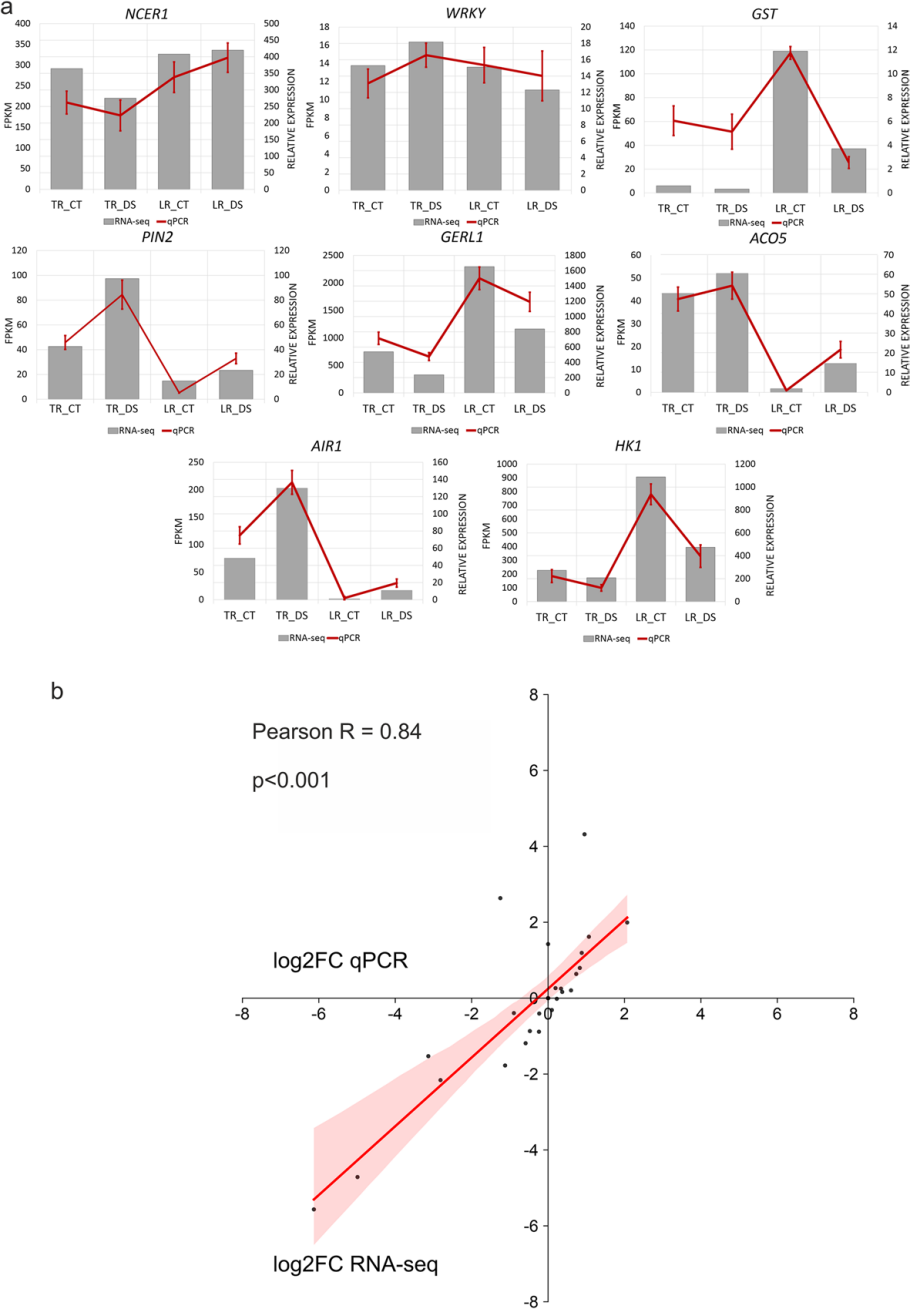
Correlation between gene expression levels obtained from RNA-seq and RT-qPCR analyses: (**a**) Graphical representation demonstrating consistent expression trends for individual genes as determined by both RNA-seq and RT-qPCR; (**b**) Pearson correlation analysis based on log2FC values, comparing gene expression levels derived from RNA-seq with those quantified by RT-qPCR. The correlation plot highlights the strong agreement between the two methodologies. *NCER1 – neutral ceramidase 1*; *WRKY* – *WRKY transcription factor; GST* – *glutathione S-transferase; PIN2* – *auxin efflux carrier component 2*; *GERL1* – *germin-like protein 1*; *ACO5* – *1-aminocyclopropane-1-carboxylate oxidase 5*; *AIR1* – *lipid-binding protein AIR1*; *HK1* – *hexokinase-1-like*; TR – taproot; LR – lateral root; CT – standard condition; DR – drought stress

To confirm the RNA-seq results, RT-qPCR was carried out on 8 genes, randomly selected from the common genes, using the same RNA samples as in the RNA-seq analysis. A total of 4 RNA-seq samples were validated by qRT-PCR (8 representative genes in 1 biological replicate and 3 technical replicates for every different types of taproot or lateral roots (Fig. 8a). Correlation of the expression ratios from the RNA-seq and qRT-PCR data were highly correlated (R = 0.84, p < 0.001) (Fig. 8b).

### Distinct hormonal adjustments in taproots and lateral roots under drought stress

**Fig. 9:**
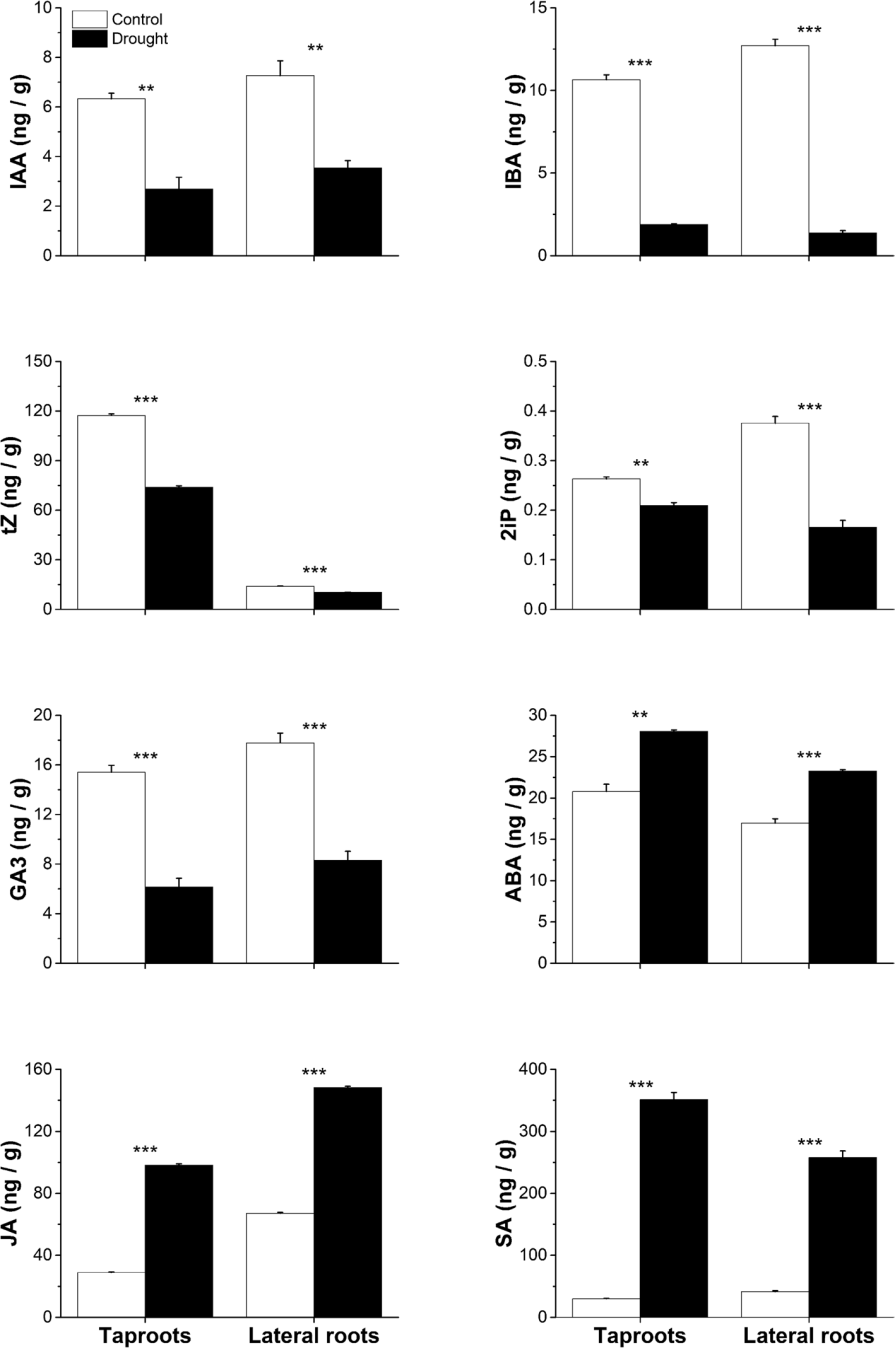
Endogenous hormone concentrations in the meristematic zone of taproots and lateral roots in pedunculate oak *(Quercus robur* L.) cuttings under well-watered conditions (white bars) and drought stress (black bars). Asterix denote statistically significant differences between treatment means within each root type as determined by Student's t-test at **P < 0.01 and ***P < 0.001. Error bars represent standard error of the mean. IAA (indole-3-acetic acid); IBA (indole-3-butyric acid); tZ (trans-zeatin); 2iP (isopentenyladenine); GA3 (gibberellin 3); ABA (abscisic acid); JA (jasmonic acid); SA (salicylic acid)

We observed that ABA levels increased by higher degree (35%) under drought stress TR-DS and by 37% in LR-DS roots. Similarly, JA, and SA levels increased under drought stress in both TR-DS by 239% and 1072% respectively and LR-DS 120% and 522% respectively. In contrast, the levels of IAA, IBA, 2iP, tZ and GA3 decreased under drought stress in both TR-DS and LR-DS, however the IBA and 2iP drop was lower in TR-DS and IAA, tZ and GA3 was lower in LR-DS. These findings highlight distinct hormonal responses to drought stress in taproots and lateral roots (Fig. 9).

## Discussion

The expression patterns of the transcriptome and drought-mediated functional profiles of miRNA reveal distinct implications for diversity within the root system, contributing to trees' resistance to drought. Notably, seedlings initially grown in containers and later transplanted to rhizotrons before drought stress exposure exhibited some physiological and molecular differences compared to acorn-sown controls. This likely reflects their early containerized growth phase, which imposes distinct developmental constraints on root architecture. Importantly, our results demonstrate that taproots and lateral roots adopt different programs when coping with drought: taproots prioritize metabolic resilience and sustained growth, whereas lateral roots emphasize growth restriction and structural adaptation. This strategic divergence underlines the necessity to consider both root types when evaluating drought tolerance in oak seedlings.

The significance of diversification within root system has been recognized in DEG expression that may improve oaks tolerance to drought stress. DEG analysis in the taproot revealed numerous genes encompassing a wide range of functions, from direct responses to drought stress (e.g., dehydrins, LEA proteins, osmoprotectants), to the stabilization of proteins and cellular structures (e.g., heat shock proteins, ubiquitin), hormonal regulation (e.g., ABA, auxins, ethylene, cytokinins) that coordinates the stress response, detoxification of reactive oxygen species (ROS) and other harmful compounds (e.g., cytochrome P450s, peroxidases, glutathione S-transferases). Briefly increased expression of genes encoding transcription factors (e.g., transcription factor BEE 1-like, transcription factor ICE1-like) provide together with decrease in Fo/Fm, Fv/Fm, and Fv/Fo strong evidence of drought stress in the analyzed plants. Under a such conditions elevated expression of genes related to carbohydrate metabolism (e.g., beta-glucosidase 12-like, inositol oxygenase), involved in glycoside and inositol metabolism are necessary to improve an osmoprotective function. Additionally, an increased proportion of genes encoding enzymes involved in cell wall modification (e.g., xyloglucan endotransglucosylase/hydrolase protein 10, polygalacturonase) together with the modulation genes engaged in resource utilization under stress conditions [28] are attributed to enhanced cell wall flexibility during taproot elongation to rise water acquisition potential [45]. Further support for genes involvement in enhancing plant resistance against water shortage can be find in GO analysis results associated with cell wall modification and cell elongation due auxin signaling in taproots, whereas more intensive presence of genes associated with ABA and brassinosteroids in lateral roots together with presence of genes involved in detoxification of ROS should be associated with stronger experience of stress in leaves (ABA) and reducing root elongation (ethylene) in favor of increasing branching of lateral roots (brassinosteroids). These findings highlight that taproots, by maintaining metabolic activity and growth, may support deeper water acquisition, while lateral roots, through growth inhibition and increased branching, may be more adaptive to fluctuating moisture due to periodic rainfall in upper soil layers. This strategic differentiation is especially relevant for optimizing nursery practices and improving drought resilience in reforestation efforts.

Under drought stress, taproots (TR-DS) exhibit stronger metabolic adaptation (enhanced carbohydrate and glutathione metabolism) and maintain growth potential (smaller decline in IBA/2iP, activation of miR396a/miR167a), whereas lateral roots (LR-DS) prioritize survival through growth suppression (sharper decrease in IAA/GA3/tZ, dominance of growth-inhibiting miRNAs) and cytoskeletal reorganization, reflecting their distinct strategies: TR-DS – tolerance with sustained metabolic function, LR-DS – resistance via growth restriction. Indeed, KEGG pathway analysis of lateral roots also showed that the enrichment of pathways associated with motor proteins, pyruvate metabolism, and the TCA cycle indicates that lateral roots are also actively adapting to stress, albeit in a slightly different manner than taproot [46, 47]. Contrary in taproot the increased expression of genes involved in processes such as motor proteins may reflect enhanced transport of nutrients and metabolites within cells, which is crucial under stress conditions. The upregulation of pyruvate metabolism suggests increased energy production and precursors for the biosynthesis of compounds essential for stress survival, confirmed in the enrichment of pathways related to starch and sucrose metabolism, glycolysis/gluconeogenesis, glutathione metabolism, motor proteins, pyruvate metabolism, and the TCA cycle suggests increased metabolic activity and defense mechanisms against drought stress [48, 49].

The upregulation of pathways related to starch and sucrose metabolism may help maintain osmotic pressure in cells, preventing water loss [50]. The enrichment of the glycolysis/gluconeogenesis pathway suggests that the plant increases energy production and metabolic precursors to cope with stress. Similarly, the significant increase in genes associated with glutathione metabolism indicates enhanced detoxification of reactive oxygen species (ROS), protecting cells from oxidative damage [51]. Finally, the enrichment of the citrate cycle (TCA cycle) indicates heightened energy production and metabolites necessary to maintain cellular functions under stress conditions [51]. Summarizing drought stress, taproots (TR) prioritize metabolic resilience through enhanced starch/sucrose metabolism, glycolysis, and glutathione pathways, while lateral roots (LR) focus on structural survival via cytoskeletal and chromatin adjustments, reflecting a trade-off between metabolic adaptation (TR) and growth-limiting resistance (LR). Notably, seedlings initially grown in containers and later transplanted to rhizotrons before drought stress showed some miRNA-mediated responses compared to acorn-sown controls, reflecting legacy effects of their early cultivation conditions. Similar to DEG and KEGG, miRNA also exhibited differential responses in taproot and lateral roots under drought stress. The up-regulation of miRNAs such as miR172, miR164, miR166, and miR393 in taproots of containerized seedlings suggests the activation of pathways related to auxin mediated root system growth and development, optimization of water management, and protection against senescence [52]. The observed downregulation of growth-promoting miRNAs (e.g., miR166b, miR171e) in these plants indicates containerization may impose constraints on meristem reactivation post-drought. Simultaneously, the down-regulation of miRNAs in taproots, including miR166b, miR171e, and miR319a, may influence root architecture modulation to inhibit new lateral root emergence and the inhibition of aboveground growth, promoting water conservation [53, 54]. In lateral roots, up-regulation of miRNAs such as miR171, miR393, miR166, and miR162 suggests the activation of pathways associated with e.g. lateral roots growth due to their branching as drought response [52]. Conversely, the down-regulation of miRNAs, including miR156a, miR166f, and miR171c, may lead to decreased osmoprotection but also structural and physiological changes that enhance tolerance to water shortage [55]. Their differential expression suggests a specialized role in taproots for stress adaptation and growth regulation, as up-regulation of these miRNAs in lateral roots likely supports their role in modulating root architecture and stress responses in this tissue. The novel miRNAs, while not yet fully characterized, are likely to contribute to similar pathways based on their co-expression with known stress-responsive miRNAs. Moreover, the expression levels of five exemplary miRNAs and their corresponding target genes – including key regulatory interactions for root development (miR166-ATHB15/REV controlling xylem differentiation), growth regulation (miR396-GRF4/10 modulating meristem activity), and auxin response (miR167b-ARF6 influencing lateral root formation) under drought stress – highlight the complex nature of miRNA–target gene regulation. These interactions demonstrate how tissue-specific expression patterns and additional regulatory mechanisms likely influence drought responses. Notably, target gene expression may not be controlled solely by miRNAs, as evidenced by the varied correlation patterns observed between these miRNA-target pairs. Other factors, such as miRNA sponges or alternative regulatory pathways, could also play important roles in modulating these relationships during drought stress.

We observed a close relationship between miRNAs and the concentration of several hormones. An enhanced concentration of ABA co-occurred with miR319 downregulation in both taproots and lateral roots, suggesting that these tissues not only regulate stomatal closure during drought stress, but also contribute to growth suppression. The stronger decrease in miR319 expression in lateral roots (− 1.73) compared to taproots (− 0.96) indicates that lateral roots experienced a more severe stress response. Moreover, the inverse relationship between ABA accumulation and miR164 downregulation observed in both root types likely represents a conserved drought adaptation strategy, as evidenced by similar patterns reported in wheat seedlings. [56–59]. Notably, the JA/SA–miR393 regulatory module forms a positive feedback loop: while JA induces defense gene expression, it also promotes the accumulation of miR393, which was over three times higher in lateral roots compared to taproots (4.13 vs. 1.16, respectively). miR393 attenuates auxin signaling, thereby suppressing lateral root growth and reinforcing a drought-adaptive phenotypic shift [60, 61]. On the other hand, growth-related hormones, such as IAA, GA3, IBA, 2iP, and tZ, show a stronger decrease in lateral roots, indicating the plant's prioritization of survival over growth. The reduction in auxins (e.g., IAA, IBA), gibberellins (e.g., GA3) and cytokinins (e.g., 2iP, tZ) suggests suppression of growth-related processes, redirecting energy toward stress adaptation and survival mechanisms [13, 57, 62, 63].

These hormonal-miRNAs adjustments enable the plant to optimize its physiological responses to drought stress, balancing growth inhibition with the stimulation of specific root types, while activating stress defense and resource conservation strategies. The interplay between ABA, JA, SA, ethylene, and the downregulation of growth-promoting hormones like auxins, gibberellins, and cytokinins highlights the complexity of the plant's hormonal network in adapting to adverse environmental conditions.

## Conclusion

This study elucidates the multifaceted mechanisms by which taproots and lateral roots coordinate drought responses in containerized oak seedlings that differ from acorn-sown cultivation techniques. Containerized plants exhibited trade-offs between metabolic adaptation (taproots) and growth arrest (lateral roots), with miRNA-mediated hormonal shifts (e.g., miR393-auxin suppression) likely affecting root regrowth potential post-stress. The degradome analysis revealed tissue-specific miRNA targeting patterns. It confirmed classical inverse correlations, such as miR166 targeting ATHB15, but also identified unexpected co-upregulation cases, like miR167b targeting ARF6. On the other hand considering stronger metabolic adaptation (enhanced carbohydrate and glutathione metabolism) and maintained growth potential (smaller decline in IBA/2iP, activation of miR396a/miR167a), along with an increased proportion of genes encoding enzymes involved in cell wall modification (e.g., xyloglucan endotransglucosylase/hydrolase protein 10, polygalacturonase) and the modulation of genes engaged in resource utilization under stress conditions, taproots of containerized seedlings are attributed to enhanced cell wall flexibility during elongation, increasing water acquisition potential. This suggests that containerized seedlings may be some alternative for acorn-sown cultivation techniques. The enrichment of stress-related pathways (e.g., glutathione metabolism) and repression of growth hormones (e.g., IAA, GA3) highlight how cultivation techniques in nursery practices (e.g., containerization) may amplify drought-induced physiological trade-offs, potentially affecting field performance after outplanting. In summary, our findings clearly differentiate the strategic roles of taproots and lateral roots under drought: taproots act as a metabolic backbone supporting water uptake and growth, while lateral roots provide adaptive plasticity through structural changes and stress signaling. This knowledge can enhance forest management practices, particularly the optimization of container seedling production to enhance drought resilience and post-planting survival in changing climate scenarios. This is important because the oak root system—comprising a taproot and lateral roots—should function as a system of communicating vessels. Consequently, the intended goal of nursery practices, which is to promote lateral root proliferation in container cassettes, not always results in enhanced resistance to drought. This is particularly evident when the taproot of containerized oaks fails to regenerate after planting, or develops more weakly than in acorn-sown oaks. Despite this research provides a foundation for developing science-based strategies to improve oak resilience to climate change, offering critical insights into the molecular regulation of drought adaptation in forest trees, similar research should be extended to trees growing in the field.

### Practical implications

Our findings provide actionable insights for forest management and nursery practices. The clear differentiation between taproot and lateral root drought strategies suggests that optimizing container seedling production should focus not only on profound growth of lateral root absorbing water from shallow soil layers but also on promoting post-planting taproot re-establishment in field-grown seedlings that will enable enhanced water uptake and metabolic resilience. These results indicate that containerized seedlings with well-developed taproots may exhibit improved drought tolerance and post-planting survival, especially under projected climate change scenarios when desiccation of surface soil horizons hinders water availability to plants. Engagement molecular and physiological screening for root system traits into nursery protocols could enhance the selection and production of oak seedlings better adapted to future environmental stresses. Ultimately, this research supports the development of science-based guidelines for reforestation and afforestation programs, contributing to the long-term sustainability and resilience of forest ecosystems.

## Supplementary Information

Supplementary Material 1.

Supplementary Material 2.

Supplementary Material 3.

Supplementary Material 4.

Supplementary Material 5.

Supplementary Material 6.

Supplementary Material 7.

Supplementary Material 8.

Supplementary Material 9.

Supplementary Material 10.

## Data Availability

The transcriptome, miRNA, and degradome sequencing data generated and analyzed in this study are available in the NCBI GEO database under the following accession numbers: GSE295319 for the transcriptome, GSE295320 for the miRNA dataset, and GSE295322 for the degradome.

## Abbreviations

TR: Taproots
LR: Lateral roots
TR-CT: Meristematic zone of taproots under control conditions
LR-CT: Lateral roots under control conditions
TR-DS: Meristematic zone of taproots under drought stress
LR-DS: Lateral roots under drought stress
