# Supplementary figures and images for: Oaks drought-induced responses under root types: gene and microRNA cooperation
Source: BMC Plant Biol. 2025 Oct 1;25:1262. doi: 10.1186/s12870-025-07402-z (PMC12490147; doi:10.1186/s12870-025-07402-z)

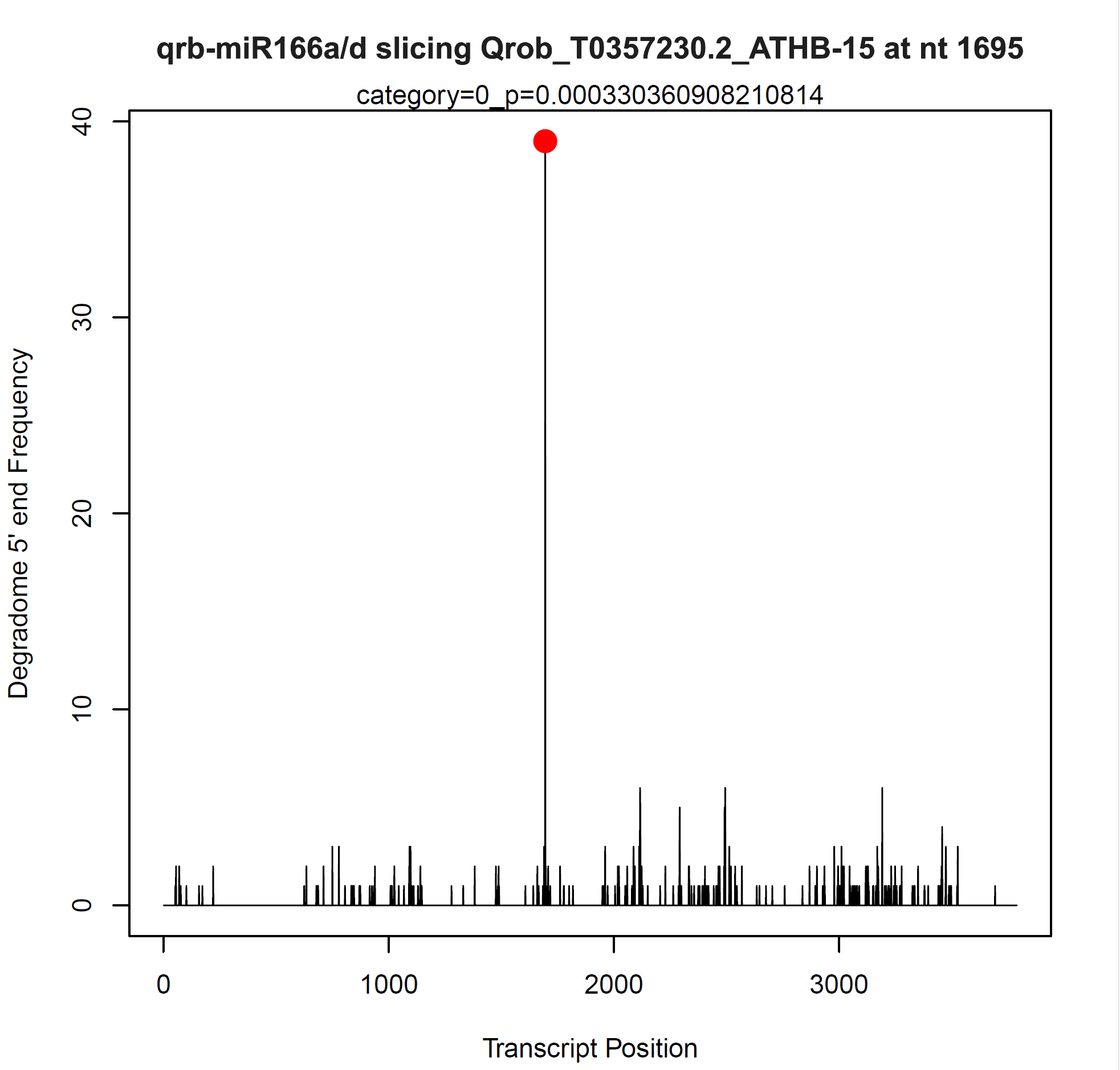

Supplement: Supplementary file 1 — Supplementary Material 1. [file 12870_2025_7402_MOESM1_ESM.png]

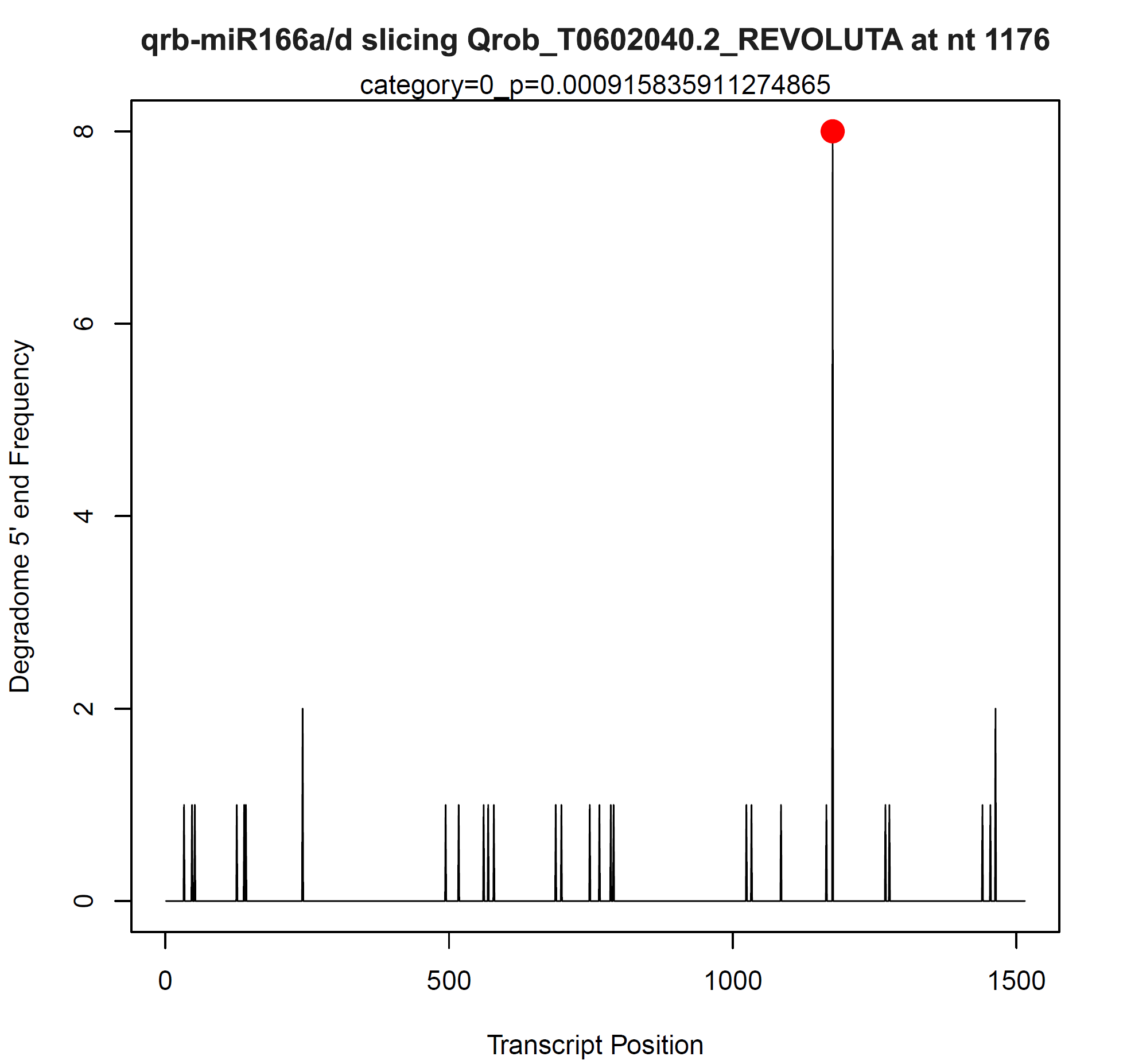

Supplement: Supplementary file 2 — Supplementary Material 2. [file 12870_2025_7402_MOESM2_ESM.png]

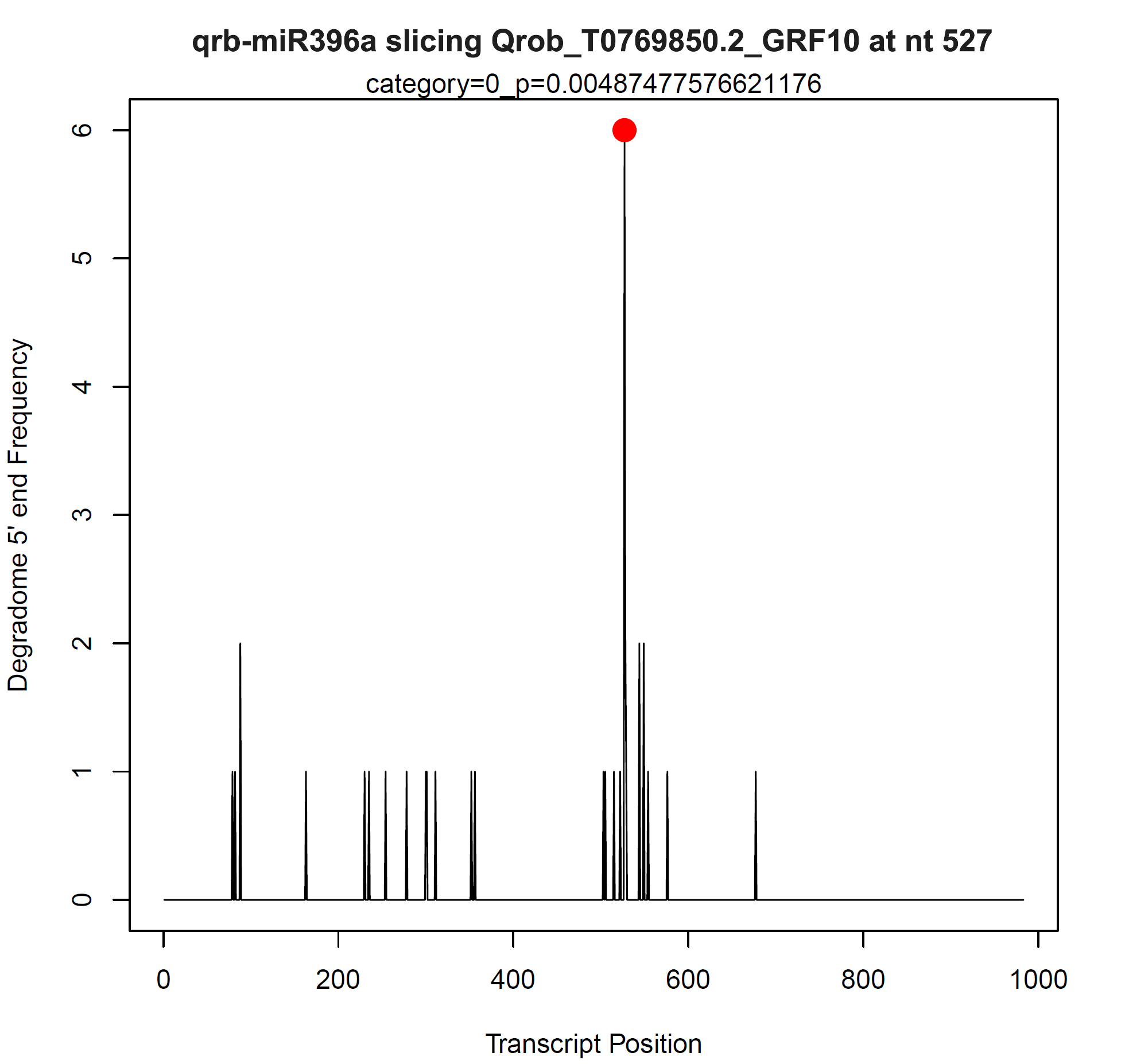

Supplement: Supplementary file 3 — Supplementary Material 3. [file 12870_2025_7402_MOESM3_ESM.png]

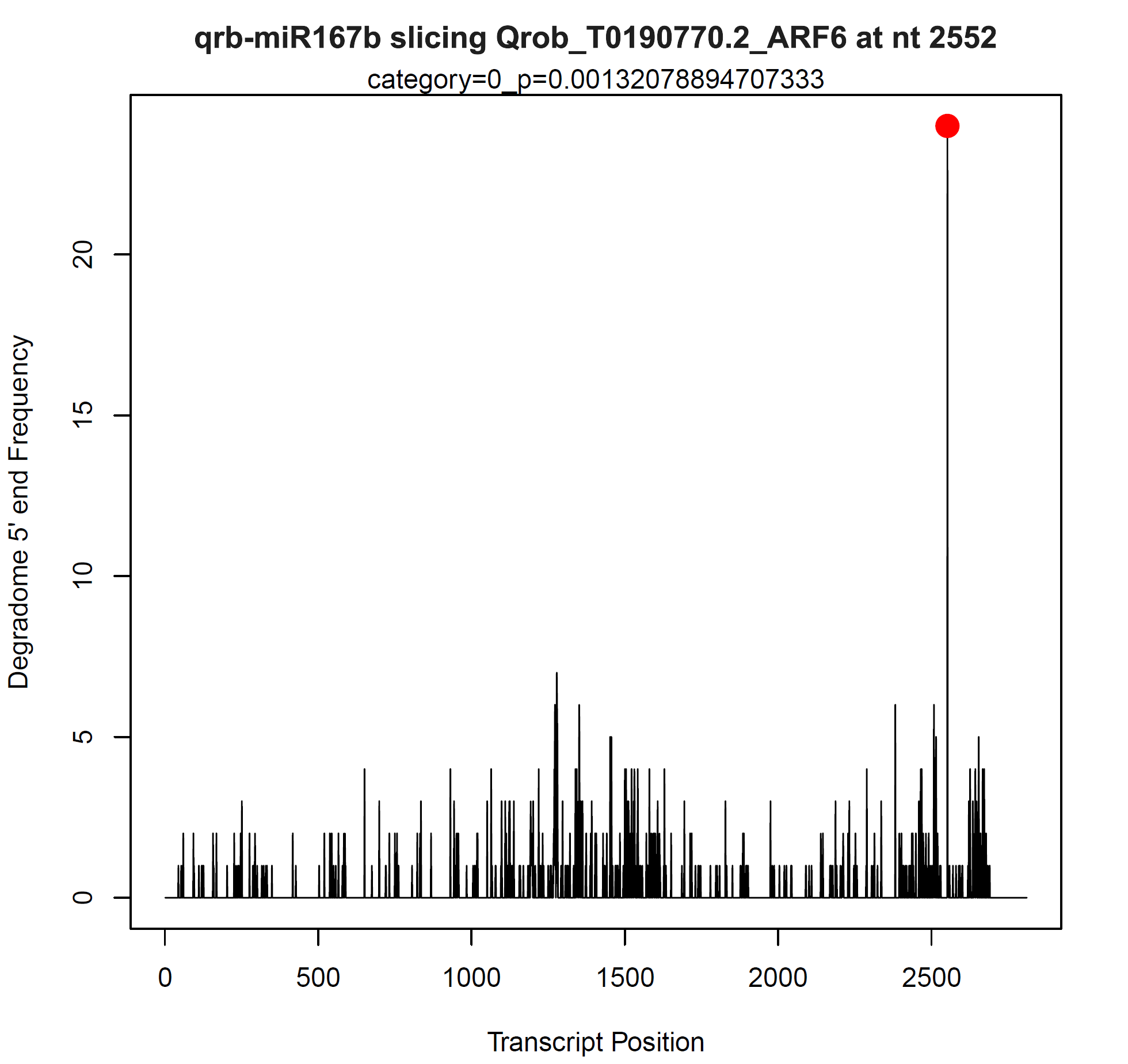

Supplement: Supplementary file 4 — Supplementary Material 4. [file 12870_2025_7402_MOESM4_ESM.png]
